# Use of a multi-method approach to rapidly assess the impact of public health policies at the state and local level: a case study of flavored e-cigarette policies

**DOI:** 10.1186/s12889-023-15408-1

**Published:** 2023-04-04

**Authors:** Elizabeth L. Seaman, Jennifer Kreslake, Katrina F. Trivers, Fatma Romeh M. Ali, Jamie Cordova, Sarah Mills, Bidisha Sinha, Brian King, Donna Vallone

**Affiliations:** 1 CDC Foundation, Atlanta, GA USA; 2 Truth Initiative, Washington, DC USA; 3 Office on Smoking and Health, National Center for Chronic Disease Prevention and Health Promotion, Centers for Disease Control and Prevention, Atlanta, GA USA

**Keywords:** Policy evaluation methods, Rapidly-available data, E-Cigarettes

## Abstract

**Background:**

E-cigarettes are the most-commonly used tobacco product by youth since 2014. To prevent youth access and use of e-cigarettes, many U.S. states and localities have enacted policies over a relatively short period of time. The adoption of these policies has necessitated timely data collection to evaluate impacts.

**Methods:**

To assess the impact of flavored e-cigarette policies in select states and local jurisdictions across the United States, a multi-method, complementary approach was implemented from July 2019 to present, which includes analyses of cross-sectional online surveys of young people ages 13–24 years with retail sales data.

**Results:**

From February 2020 through February 2023, cross-sectional surveys have been conducted in three cities, one county, and eight states where policy changes have been enacted or are likely to be enacted. Data collection occurred every six months to provide near real-time data and examine trends over time. Additionally, weekly retail sales data were aggregated to showcase monthly sales trends at the national level and for the selected states.

**Discussion:**

This rapid and efficient method of coupling online survey data with retail sales data provides a timely and effective approach for monitoring a quickly changing tobacco product landscape, particularly for states and localities where rapidly-available data is often not available. This approach can also be used to monitor other health behaviors and relevant policy impacts.

## Introduction

E-cigarettes entered the United States (U.S.) marketplace in 2007. Since 2014, e-cigarettes have been the most commonly used tobacco product among U.S. high school and middle school students, with 14.1% (2.14 million) of high school students and 3.3% (380,000) of middle school students reporting current use in 2022 [1]. E-cigarettes are available in a variety of flavors which appeal to youth and young adults; in 2022, 84.9% of high school e-cigarette current users reported using flavored products [1]. The use of e-cigarettes among youth is a public health concern; nicotine is highly addictive and can harm an adolescents’ developing brain [2]. Additionally, the use of e-cigarettes among youth may lead to cigarette smoking [3].

In response to prevalent e-cigarette use among youth across the U.S., as of December 2022, eight states and over 370 localities have enacted some type of restriction on flavored tobacco product sales [4]. Policies that prohibit the sale of tobacco products vary greatly in terms of included products and flavors, which can influence the strength of these policies for impacting public health [5]. For example, some policies prohibit the sale of all flavors, whereas others exempt menthol flavored products. Moreover, some focus on all tobacco products, while others focus only on e-cigarettes.

Prior research has analyzed the impact of flavored e-cigarette restriction policies specifically [6–9], but much of this work has been limited to one location (e.g., one state or one locality) using one methodology (e.g., surveys, retail environment scans), which provides assessed short-term impacts, or did not assess the impact of the policy on youth and young adult access and use specifically. The result of these analyses generally demonstrate that these policies are effective when adequately enforced; however, impact varies depending on the strength of the policy, as well as the extent of retailer enforcement. For example, compliance with restrictions was high in Massachusetts [8], and inventories of restricted products declined in California communities [7]. Moreover, higher perceived difficulty of accessing flavored products was reported in California [6]. Conversely, while overall e-cigarette use continued to decline among youth in New York State following policy implementation, some continued to access and use restricted flavors, highlight opportunities for increased compliance [9]. Actions have also been taken by the tobacco industry in response to these policies in attempt to prevent their enactment or to mitigate their intended effects [10]. Employing relevant and timely data collection and analysis across states and localities with various restriction policies is essential to assess behavioral responses to these various policies, which can help inform future policy development and implementation at the national, state, and local levels.

The “Monitoring E-Cigarette Use Among Youth in Select U.S. Cities and States” project was designed to assess real-time impacts of state and local-level flavored e-cigarette policies. The project uses two types of data: online cross-sectional surveys and retail sales data. The online cross-sectional surveys provide individual-level responses for tobacco-related knowledge, attitudes, and behavior, while the weekly sales data reflects point-of-sale transactions for all tobacco products which provides a market view of the tobacco product landscape. This paper describes this multi- method approach and how it might be employed by researchers for evaluating public health policies at the national, state and local levels.

## Materials and methods

### Online cross-sectional surveys

#### Data description

As part of the “Monitoring E-Cigarette Use Among Youth in Select U.S. Cities and States” project, a series of repeated, cross-sectional online surveys were developed by the CDC Foundation and project partners and administered, using convenience sampling, beginning in February 2020. The surveys were conducted by Ipsos Public Affairs LLC, one of the largest market research and polling companies globally. The surveys were administered to youth and young adults ages 13–24 years. The data collection timeline was designed to allow for comparison of pre- and post- policy implementation where possible. Timing of related to phases of implementation must be accounted for when evaluating the impact of any policy. Table 1 provides a list of the selected states and localities, specific data collection periods and site-specific policies, including effective date of the policy. Of note, after a policy is enacted by state or local officials, it might not become effective until a future date, which is typically to allow time for retailers and others to be educated about the policy and to become compliant. For this analysis, the effective date was the primary standard used to evaluate impact.

**Table 1:** Survey sites and fielding

	Survey 1		Survey 2		Survey 3		Survey 4		Survey 5		Survey 6		Site Policy Effective Date
			Approximately 6 Months from Baseline Fielding Start		Approximately 12 Months from Baseline Fielding Start		Approximately 18 Months from Baseline Fielding Start		Approximately 24 Months from Baseline Fielding Start		Approximately 30 Months from Baseline Fielding Start		
	Fielding Start	Fielding End	Fielding Start	Fielding End	Fielding Start	Fielding End	Fielding Start	Fielding End	Fielding Start	Fielding End	Fielding Start	Fielding End	
**A Sites**													
Massachusetts	2/26/2020	4/8/2020	8/26/2020	10/30/2020	2/26/2021	5/7/2021	9/24/2021	1/5/2022	7/8/2022	10/7/2022	~ 2/8/2023		9/25/2019 -12/11/2019
													11/27/2019
													6/1/2020
Los Angeles City	2/26/2020	4/8/2020	8/26/2020	11/13/2020	2/26/2021	5/7/2021	9/24/2021	1/5/2022	7/8/2022	10/12/2022	~ 2/8/2023		N/A
Los Angeles County	2/26/2020	4/8/2020	8/26/2020	11/13/2020	2/26/2021	5/13/2021	9/24/2021	1/4/2022	7/8/2022	10/17/2022	~ 2/8/2023		5/1/2020
New Jersey	2/26/2020	4/8/2020	8/26/2020	10/27/2020	2/26/2021	5/7/2021	9/24/2021	12/7/2021	7/8/2022	10/3/2022	~ 2/8/2023		4/20/2020
New York City	2/26/2020	4/8/2020	8/26/2020	10/27/2020	2/26/2021	5/7/2021	9/24/2021	12/28/2021	7/8/2022	10/7/2022	~ 2/8/2023		7/1/2020
**B Sites**													
California (less LA City and LA County)	4/15/2020	5/27/2020	11/13/2020	1/4/2021	5/26/2021	7/26/2021	11/29/2021	2/14/2022	7/8/2022	9/23/2022	~ 2/8/2023		12/21/2022
New York (less New York City)	4/15/2020	5/27/2020	11/13/2020	1/4/2021	5/26/2021	7/26/2021	11/29/2021	2/14/2022	7/8/2022	9/23/2022	~ 2/8/2023		5/18/2020
**C Sites**													
Illinois *(less Chicago)*	8/6/2020	10/8/2020	2/26/2021	5/7/2021	9/24/2021	12/10/2021	3/25/2022	5/12/2022	9/26/2022	12/6/2022	~ 3/25/2023		N/A
Chicago	8/6/2020	10/8/2020	2/26/2021	5/21/2021	9/24/2021	1/5/2022	3/25/2022	6/9/2022	9/26/2022	12/28/2022	~ 3/25/2023		10/7/2020
Arizona	8/6/2020	10/8/2020	2/26/2021	5/7/2021	9/24/2021	1/4/2022	3/25/2022	6/8/2022	9/26/2022	12/28/2022	~ 3/25/2023		N/A
Colorado	8/6/2020	10/8/2020	2/26/2021	5/7/2021	9/24/2021	12/1/2021	3/25/2022	6/2/2022	9/26/2022	12/28/2022	~ 3/25/2023		N/A
**Additional Sites**													
North Carolina	2/26/2021	4/16/2021	3/30/2022	5/24/2022									N/A

Study-specific post-stratification weights were developed after each survey by the survey research firm to ensure that the sample of respondents was representative of the population in the area of interest. Specifically, each site’s sample was compared to the U.S. Census and then weights were created to ensure representativeness. Separate weights were created for each site for each round of data collection. Comparisons were limited to within sites over time and not across sites – data from New York City could not be directly compared to data from Los Angeles City, for example.

##### Power and sample size calculations

Power calculations were conducted to ensure sufficient sample to detect significant changes. An a priori power calculation was performed to allow for a 5% minimum detectable difference with a point estimate of 15%, with 80% power and a two-tailed significance level of 0.05, which yielded a minimum sample size of 943 respondents per site per survey.

##### Site selection

States and localities were selected based on a review of areas that had implemented, or were likely to implement, flavored e-cigarette policy initiatives based on feedback from partners knowledgeable about state and local policy and legislation efforts. Sites were prioritized based on likelihood of policy passage and grouped into “simultaneous cohorts” to be launched. The “A” cohort of sites included New York City, City of Los Angeles, unincorporated parts of Los Angeles County, Massachusetts and New Jersey. The “B” cohort of sites included California, excluding City of Los Angeles and Los Angeles County, and New York state, excluding New York City. The “C” cohort of sites included the city of Chicago, Illinois (excluding the city of Chicago), Colorado and Arizona.

##### Survey instrument

The online cross-sectional survey instrument included numerous validated items to assess tobacco product (e-cigarettes, cigarettes, cigar products, hookah/waterpipe, smokeless products, heated products) use, knowledge, attitudes, beliefs and behaviors, risk perceptions, product purchase patterns, sociodemographic factors and other substance use. Tobacco use questions included ever use, age of initiation, reason for use, current (past 30-day) use, use of flavored products, brands used, product access source and cessation behavior. Each tobacco product section had a preamble with a written description of the product and example images. Items were drawn from established data collection efforts, including the National Youth Tobacco Survey (NYTS), the Population Assessment of Tobacco and Health (PATH), and the Truth Longitudinal Cohort (TLC) surveys. In addition, several novel items were developed to assess emerging products or topics that had not been previously studied in existing surveys. The final survey was approximately fifteen to twenty minutes in duration. Table 2 provides a detailed description of survey domains and constructs.

**Table 2:** Survey domains and constructs

Domain	Construct
**Demographics**	Gender
	LGBT identity
	Age
	Race/Ethnicity
	Languages spoken
	Education - highest degree received
	Current educational enrollment
	Household income
	Marital status
	Live with partner
	Head of household
	Household size
	Employment status
	Housing type
	Family financial situation
**E-Cigarettes**	Ever use
	Age of initiation
	Reason for use
	Past 30-day frequency of use
	E-cigarette device type
	Use of flavored products
	Brands used in past 30-days
	Product access source
	Product access source (e-liquid/cartridge/refill)
	Time to first e-cigarette
	Cessation behavior (intentions, attempts, methods)
	Susceptibility to use in next year
**Cigarettes**	Ever use
	Age of initiation
	Past 30-day frequency of use
	Past 30-day intensity of use
	Past 30-day use of menthol cigarettes
	Cessation behavior (intention, attempts)
	Susceptibility to use in next year
**Cigar Products (Large Cigars, Small Cigars, Cigarillos)**	Ever use
	Age of initiation
	Reason for use
	Past 30-day frequency of use
	Past 30-day intensity of use
	Use of flavored products
	Cessation behavior (intention, attempts)
	Susceptibility to use in next year
**Hookah**	Ever use
	Age of initiation
	Reason for use
	Past 30-day frequency of use
	Use of flavored products
	Susceptibility to use in next year
**Smokeless Tobacco and Snus**	Ever use
	Past 30-day frequency of use
	Use of flavored products
	Cessation attempts
**Heated Tobacco Product Use**	Ever use
	Past 30-day use
**Other Substance Use**	Synthetic nicotine product awareness
	Past 30-day heavy/binge drinking^a^
	Ever use of marijuana
	Past 30-day marijuana use frequency
	Mode of marijuana use
**Environment**	Tobacco product harm perceptions
	Tobacco product addictiveness perceptions
	Attitudes towards e-cigarettes
	Household exposure to tobacco products
	Social media utilization
	Video streaming utilization
	Exposure to e-cigarette advertising
	Receipt of e-cigarette coupons
	COVID-19 related behaviors^b^

**Footnotes**

**a**: In this survey, heavy/binge drinking refers to having 5 or more alcoholic drinks on an occasion.

**b**: Respondents were asked about COVID-19 related behaviors including social distancing and how their e-cigarette use/attitudes/beliefs had or had not been impacted.

##### Recruitment

The survey leveraged respondents from Ipsos’ KnowledgePanel®, a probability-based online panel, as well as a newly-recruited participants through convenience sampling methodologies. The survey research firm worked with survey sampling vendors to invite eligible participants to complete the online survey. Respondent eligibility was assessed by online age verification and residential zip code to ensure respondents met inclusion criteria of being between 13 and 24 years old and residing in the targeted location. Participants received incentives in the form of nominal reward points which can be redeemed for awards. Generally, the incentive for completing the survey was between 10,000 and 15,000 points, which roughly equates to $10 to $15.

##### Consent

All consent was obtained online. For youth participants (ages 13–17 years), parents were asked to provide parental consent and youth were asked to provide their own assent to participate. Young adult (ages 18–24 years) participants provided consent to participate.

#### Survey data collection schedule

Surveys were conducted every six months to maximize the capacity to detect relatively short-term changes in outcomes of interest against the context of a quickly changing policy and product landscape. Since survey data collection typically required approximately six to twelve weeks to complete, data collection every six months allowed sufficient time for recruitment and analysis prior to the next data collection period. Once data collection was completed, a comprehensive set of data quality control and data cleaning efforts were performed. Unfortunately, participation was significantly impacted by the COVID-19 pandemic, which resulted in longer survey fielding periods. The survey vendor reported that online survey participation slowed during the COVID-19 pandemic across surveys; there are several potential reasons for this phenomenon that the survey vendor’s survey methodology team hypothesized, including respondent survey fatigue and competing priorities among respondents. Initially, survey fielding periods were projected to be 6 weeks, but over time survey fielding time was closer to eight to twelve weeks depending on site and survey (Table 1).

#### Analysis plan

##### Data analyses and dissemination

Descriptive summary tables of sociodemographic factors and tobacco use were developed for each phase of survey data collection across sites. Statistical tests including t-tests and analysis of variance (ANOVA) were used to assess associations between the timing of policy implementation and changes in tobacco-related knowledge, attitudes, beliefs and behaviors. There are plans to disseminate empirical findings through future manuscripts.

### Retail sales data

The retail sales data was purchased from Information Resources, Inc. (IRI) (www.iriworldwide.com/en-US), a major commercial aggregator of retail sales data. This product-level retail sales data was analyzed monthly to assess market-level changes in all tobacco product sales. For this project, the focus was on examining changes in e-cigarette product sales in relation to flavored e-cigarette policy implementation.

#### Data description

Retail sales data reflects tobacco product sales registered through the electronic scanning of the Universal Product Code (UPC), a barcode symbology that is widely used for tracking trade items in stores (11). The data included sales from convenience stores, gas stations, grocery stores, drugstores/pharmacies, mass merchandiser outlets, club stores, dollar stores, and military sales. Sales data were not available for online sales or vape or tobacco specialty store sales. Overall, sales data were representative of the total marketplace (excluding vape shops and online sales) as a combination of Census reporting retailers (i.e., retailers who provide scanner data) and projections for non-participating retailers (12).

Efforts were required to clean the data and categorize product characteristics prior to analysis. Variables included product UPC, brand name, product name, several product features, unit sales, and dollar sales. Sales estimates are aggregated for each week by IRI and must be reported in minimum 2-week periods or more by researchers to ensure that retailers and products could not be determined from data published or posted.

#### Retail sales data schedule

For this project, available data were purchased monthly for the total United States and all available states, where releasable. Individual state-level release is dependent on adequate coverage at a channel level of census and sample stores to meet IRI’s methodology requirements for an accurate projection to total state level. Data from Alaska, Hawaii, and Montana are not commercially available. Additionally, state-level data from Delaware, Idaho, Kansas, Mississippi, Nebraska, New Jersey, and New Mexico were not examined because they did not meet IRI’s release criteria for the convenience store channel, which accounts for the majority of national tobacco sales. IRI uses proprietary calculation factors to account for non-participating retailers, yielding data that is representative of sales in the 48 continental states (excluding Alaska and Hawaii). Therefore, the national data is not the sum of state level data, and while state-level data from Delaware, Idaho, Kansas, Mississippi, Nebraska, New Jersey, and New Mexico are not reliable on an individual state-level basis, the national data is inclusive of these states as their data collectively are reliable as part of the national data.

#### Analysis plan

Summary charts illustrating trends in product units sold by flavor and device type were prepared and published monthly on the CDC Foundation website (https://www.cdcfoundation.org/programs/monitoring-e-cigarette-use-among-youth) as a part of National and State Data Briefs. These trends helped assess whether and to what extent sales varied post policy implementation.

## Results of implementation

### Online cross-sectional convenience sample surveys

As of February 2023, online surveys had been fielded in 12 sites including states, cities, and counties (Table 1). Surveys were conducted every six months with the goal of conducting at least six surveys in each site. Ideally, the surveys were timed to represent pre- and post-flavored e-cigarette policy change; however, the inherent uncertainty surrounding policy passage and enactment presented challenges with optimal timing of the surveys. Information on policy enactment was obtained following review of the text of the actual legislation as well as review of available sources from Campaign for Tobacco-Free Kids (4), Truth Initiative (13) and the Public Health Law Center (14). For example, in Massachusetts, all fielded surveys occurred after the state’s emergency action that prohibited sale of all e-cigarettes (September 2019), so all data collected was post-policy. Of the 12 survey sites, four had not passed any flavored e-cigarette policy (Illinois, Arizona, Colorado, North Carolina), one had a policy passed before survey fielding (Massachusetts), and seven had flavored tobacco policies passed between any surveys (California, City of Los Angeles, unincorporated parts of Los Angeles County, New Jersey, New York City, New York state, Chicago).

### Retail sales data dissemination

A Morbidity and Mortality Weekly Report (MMWR) was published on September 18, 2020 to present national trends in U.S. e-cigarette sales that occurred from September 2014 to May 2020 (15), including trends by product type and flavor type. Additional information about categorization and analytic methods were published in the MMWR (15) and online on the CDC Foundation’s website (16).

**Fig. 1:**
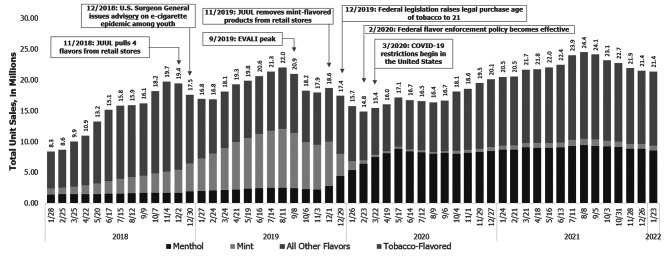
**Example sales data analysis** National E-Cigarette Unit Sales by Flavor, 4 Week Estimates 1/2017–11/2021 [17]

Sales Data Briefs that reflect national and selected state trends are regularly posted on the CDC Foundation website (https://www.cdcfoundation.org/programs/monitoring-e-cigarette-use-among-youth). Sales Data Briefs include descriptive analyses, such as total standardized unit sales overall and by flavor and product type. An example of a Sales Data Brief is presented in Fig. 1 (17).

Data Briefs for selected states where flavored e-cigarette policies have been enacted, introduced or considered are posted on the CDC Foundation website. The set of selected states include: California, Colorado, Connecticut, Illinois, Maine, Maryland, Massachusetts, Minnesota, New Hampshire, New York, Oregon, Rhode Island, Utah and Washington. Several manuscripts have been published or are underway that include analyses of these retail sales data which extend beyond the descriptive estimates presented in the Sales Data Briefs (18, 19, 20, 21, 22).

## Discussion

The value of employing this multi-method data collection and analytic approach lies in its timeliness for examining the impact of tobacco policy implementation, particularly at the subnational level for included states. Having data that is available sooner than traditional surveillance systems allows for rapid policy evaluation. Given the rapidly changing tobacco product landscape, continued efforts to monitor tobacco use behavior more frequently and efficiently, at various levels of geographic precision, is critical to informing effective policy implementation. Online data collection allows faster data collection and shorter periods of data processing as compared to telephonic or in-person surveys. Web-based surveys also facilitate the inclusion of visual imagery of tobacco products not feasible for telephone-based surveys.

These data also allow for the timely comparison of trends over time and a degree of flexibility in data collection and analysis that traditional survey methods do not permit. These data collection methods can provide time-efficient, “real world” evaluation data, which may be especially helpful for state or local health departments and policy makers who need to assess policy impacts in real time. Moreover, online data collection supplemented with sales data provides a complementary and more comprehensive picture of the tobacco epidemic than either data source alone. Figure 1 shows an example of how sales data trends can be overlaid with policy action in order to visually represent market changes on the backdrop of the policy environment. This approach can be used to monitor other health issues and policies beyond tobacco use. Retail sales data are available for virtually all consumer products (e.g., food [23], alcohol [24], sugar sweetened beverages [25]) and a cross-sectional online survey using convenience sampling methods could be tailored to a number of health topics and policy areas.

While retail sales data does not reflect the characteristics of those who purchase products (e.g., age, race/ethnicity), it does reveal overall market trends of tobacco products sold by major retailers for every month. Online surveys provide insight into tobacco product use patterns, brand preferences, and emerging issues not yet included in national surveillance surveys, particularly. items related to synthetic and non-tobacco nicotine, ice and cooling flavored e-cigarettes, and COVID-related e-cigarette behaviors. Together, these data collection efforts can provide insights into individual behavior and market-level trends in the context of policy changes at the national, state and local level.

## Limitations of this approach

Cross-sectional surveys using convenience sampling do not constitute population-level surveillance and will not supplant more rigorous methods including the gold standard of address-based probability sampling surveys. Repeated cross-sectional surveys cannot be used to assess intrapersonal trends or temporal trends like a longitudinal cohort survey would – they provide an assessment among a group of respondents at a given time. The cross-sectional nature of the data limits studying intrapersonal trends over time and comparisons are limited to within sites and not across sites. Additionally, convenience sample data may not be representative of a state or locality.

Although retail sales data reflects the majority of tobacco product sales and venues, these data do not include online and tobacco specialty/vape shop retail sales. Estimates indicate that about 20% of all e-cigarette sales are online [16]. In 2021, 2.9% of middle and high school students who used e-cigarettes in the past month purchased their e-cigarettes on the Internet, 32.3% of youth reported getting e-cigarettes from a friend and 21.5% of youth reported buying them from another person [17]. Ultimately, e-cigarettes available on the marketplace can get into the hands of youth, regardless of their initial retail source. Moreover, retail sales data provides no information about demographics or other characteristics of purchasers or consumers. Sales to youth and young adults cannot be directly assessed; however, trends in retail sales data are generally consistent with trends in use among these populations, including the marked increase documented among U.S. youth and in sales nationally during 2017–2019. Similarly, retail sales data does not correspond directly to consumption; it merely reflects products sold. Nonetheless, retail sales data, particularly when examined concurrently with survey data, may identify trends in product use behavior relative to policy implementation. This approach can provide empirical evidence to inform interventions.

Taken together, repeated cross-sectional online survey data coupled with retail sales data can provide a compelling assessment of the impact of policy implementation. Utilizing two sources of rapidly-available data has allowed for early-stage policy evaluation in the “Monitoring E-Cigarette Use Among Youth in Select U.S. Cities and States” project. This multi-method approach of harnessing rapidly available data can be particularly useful to state or local health departments to inform interventions across a diverse range of health topics and policies, including tobacco control.

## Data Availability

No empirical analyses of data are included in this article. The datasets generated during the current study are not publicly available due to a need to protect respondent privacy (survey data) and the proprietary nature of the data purchased (sales data).

## Abbreviations

ANOVA: ANalysis Of VAriance
COVID-19: Coronavirus Disease
MMWR: Morbidity and Mortality Weekly Report
NYTS: National Youth Tobacco Survey
PATH: The Population Assessment of Tobacco and Health
U.S.: United States
